# Neural Signatures of Rational and Heuristic Choice Strategies: A Single Trial ERP Analysis

**DOI:** 10.3389/fnhum.2017.00401

**Published:** 2017-08-18

**Authors:** Szymon Wichary, Mikołaj Magnuski, Tomasz Oleksy, Aneta Brzezicka

**Affiliations:** ^1^Center for Research in Economic Behavior, Wrocław Faculty of Psychology, University of Social Sciences and Humanities Wrocław, Poland; ^2^Institute of Cognitive and Behavioral Neuroscience, Faculty of Psychology, University of Social Sciences and Humanities Warsaw, Poland; ^3^Faculty of Psychology, University of Warsaw Warsaw, Poland; ^4^Department of Neurosurgery, Cedars-Sinai Medical Center, Los Angeles CA, United States

**Keywords:** decision making, heuristics, strategy selection, P3, EEG/ERP

## Abstract

In multi-attribute choice, people use heuristics to simplify decision problems. We studied the use of heuristic and rational strategies and their electrophysiological correlates. Since previous work linked the P3 ERP component to attention and decision making, we were interested whether the amplitude of this component is associated with decision strategy use. To this end, we recorded EEG when participants performed a two-alternative choice task, where they could acquire decision cues in a sequential manner and use them to make choices. We classified participants’ choices as consistent with a rational Weighted Additive rule (WADD) or a simple heuristic Take The Best (TTB). Participants differed in their preference for WADD and TTB. Using a permutation-based single trial approach, we analyzed EEG responses to consecutive decision cues and their relation to the individual strategy preference. The preference for WADD over TTB was associated with overall higher signal amplitudes to decision cues in the P3 time window. Moreover, the preference for WADD was associated with similar P3 amplitudes to consecutive cues, whereas the preference for TTB was associated with substantial decreases in P3 amplitudes to consecutive cues. We also found that the preference for TTB was associated with enhanced N1 component to cues that discriminated decision alternatives, suggesting very early attention allocation to such cues by TTB users. Our results suggest that preference for either WADD or TTB has an early neural signature reflecting differences in attentional weighting of decision cues. In light of recent findings and hypotheses regarding P3, we interpret these results as indicating the involvement of catecholamine arousal systems in shaping predecisional information processing and strategy selection.

## Introduction

When making choices, decision makers must process information, with some choices requiring information integration and others allowing for one-reason decision making. In order to decide, people have to infer the future state of the world on the basis of probabilistic cues (e.g., Rieskamp and Hoffrage, 1999). A popular theory explaining how they do it is that people have a repertoire of strategies and select them adaptively, depending on task conditions and their own dispositions (Payne et al., 1993; Gigerenzer et al., 1999). The strategies differ in how many decision cues they require and how the cues are utilized. A normative, compensatory Weighted Additive strategy (WADD) integrates all available information; it computes the sum of all cue values multiplied by their cue validities for each alternative, and selects the alternative with the largest sum (Payne et al., 1993). In contrast, a simple, non-compensatory heuristic Take The Best (TTB; Gigerenzer and Goldstein, 1996) assumes that when comparing different alternatives, the one with the highest cue value for the most valid cue is selected as the one with the highest criterion value. Together, TTB and WADD explain a high proportion of people’s multi-attribute choices in preference and inference based tasks (Payne et al., 1993; Bröder, 2000, 2003; Rieskamp and Otto, 2006; Mata et al., 2007; Rieskamp and Hoffrage, 2008; Wichary et al., 2016). In comparison to WADD users, individuals preferring TTB process information in a more selective manner, spending a greater amount of time on the analysis of the most important cue (Wichary et al., 2016). This suggest that differences in attention allocation and differential cue weighting might lead to the eventual reliance on WADD or TTB.

Do choice strategies have their distinct neural signatures? Individuals vary in the use of strategies, and several fMRI studies attempted to identify the neural sources of this variability. Venkatraman et al. (2009a), studying choices under risk, showed that individual variability in strategy use could be predicted by BOLD activity in ventral striatum, suggesting that high activity in the dopaminergic reward system might underlie the use of simplifying non-compensatory strategies. Moreover, Venkatraman et al. (2009b) provided evidence that strategic control of decisions is associated with the activity of anterior dorsomedial prefrontal cortex (DMPFC). The involvement of medial prefrontal cortex is also supported by Schuck et al. (2015), who showed that adaptive strategy switches in a perceptual decision task were associated with activity in this region. Also, Gluth et al. (2013), studying inference-based multi-attribute choice, showed the association between strategy selection and the activity of ventral striatum, the ventromedial prefrontal cortex and the anterior cingulate cortex (ACC). Khader et al. (2011), using a training paradigm in memory-based multi-attribute choice, showed that the activity of the left dorsolateral prefrontal cortex (DLPFC) reflects the number of cues required for a decision, and modulates the activity in posterior areas that store these cues.

The abovementioned studies show that several brain areas – of different functional properties – are involved in decision strategy use. However, because these studies employed fMRI which has high spatial resolution but lacks temporal precision, they were unable to track the fine grained temporal dynamics of pre-decisional brain activation. In contrast, electroencephalography (EEG) offers an opportunity to track temporal patterns of brain activity and to find early neural signatures of choice processes. One EEG signal relevant in this area is the classic P3 component of the event related potential (ERP; Picton, 1992; Verleger et al., 2005; Polich, 2007). P3 starts about 250 ms after detection of a target stimulus and is linked to attention allocation, so that motivationally significant stimuli that attract attention elicit high P3 amplitudes at parietal electrodes. Moreover, stimuli that elicit high P3 amplitudes are better encoded, suggesting that this component reflects the role of attention in memory updating and subsequent retrieval (Polich, 2014). Mechanistic explanations suggest that although P3 is generated at multiple sites in the cortex (Bledowski et al., 2004), it is modulated by the activity of dopaminergic and noradrenergic neuromodulatory systems originating in midbrain and pons, respectively. In this vein, P3 has been proposed as a reward-related electrophysiological marker (Goldstein et al., 2006; Pfabigan et al., 2014). Similarly, Nieuwenhuis et al. (2005) proposed P3 as a signal reflecting the activity of locus coeruleus-norepinephrine system (LC-NE) associated with arousal and information processing.

We extended this line of theorizing and proposed that the LC-NE activity is linked to strategy selection in multi-attribute choice. Our theoretical model (Bottom Up Model of Strategy Selection, BUMSS; Wichary and Smolen, 2016) proposes that the process of strategy selection is shaped in a bottom up manner by brain-wide gain modulation mediated by LC-NE. According to the model gain modulation coupled with lateral inhibition in the cortex, is responsible for attentional selection of some decision cues over others (Eldar et al., 2013; Warren et al., 2015; Mather et al., 2016). Following Nieuwenhuis et al. (2005) and Murphy et al. (2011), our model assumes that the physiological markers of LC-NE activity, such as P3, should be linked to pre-decisional information processing and strategy use in multi-attribute choice, because they index attention that can be preferentially allocated to relevant decision cues.

Besides P3, other EEG indices have been linked to attention allocation, at even earlier stages of information processing. The ERP component N1, a negative deflection peaking around 100 ms post-stimulus onset, has been linked to modality specific attention allocation. Particularly, N1 was proposed to reflect an early attentional mechanism employed when two stimuli must be discriminated (Vogel and Luck, 2000). It is important to note that this component features prominently in studies on perception and attention, but has not been linked to complex decision making before. However, given its links to attention allocation, it is possible that such an early EEG signal preceding P3 can also be associated with pre-decisional information processing and strategy use in multi-attribute choice.

Electroencephalography/event related potential, with the focus on P3 has been employed in studies of decision making. In studies on perceptual decision making, P3 emerged as a supramodal decision signal indexing the accumulation of perceptual evidence (O’Connell et al., 2012; Kelly and O’Connell, 2015; Twomey et al., 2015), which is consistent with Nieuwenhuis et al. (2005) hypothesis linking P3 with activity of the LC-NE system mediating cortical gain modulation (Warren et al., 2015). EEG has also been used in other decision making studies, e.g., in the context of risk taking, where P3 was found to correlate with decision making under risk and uncertainty (Zhang et al., 2014; Wang et al., 2015). In the context of heuristic strategy use in value-based effortful decision making, Achtziger et al. (2014) studied the use of the representativeness heuristic in a decision-making task and found its association with N2 and P3 amplitudes. As for multi-attribute choice based on probabilistic inference, up to date, there was only one study that employed EEG to look for neural correlates of decision strategy use in probabilistic inference tasks. Studying the use of recognition heuristic in a two-alternative choice, Rosburg et al. (2011) showed that P3 amplitude is larger to recognized decision alternatives and predicts participants’ actual choices.

In the current work, we aimed to analyze EEG/ERP correlates of the preference for simple and complex decision strategies in inference-based multi-attribute choice. We analyzed the relationships between spontaneous decision strategy use and the EEG signal amplitude to consecutive decision cues. Based on the literature linking P3 with attention allocation and based on our theoretical model of strategy selection (Wichary and Smolen, 2016), we expected that the use of the simple heuristic TTB would be associated with high P3 amplitudes to the most important decision cues and low P3 amplitudes to less important cues. In contrast, the use of WADD strategy should be associated with similar P3 amplitudes to all decision cues. Given that differences in attention processes might be indexed by earlier components, we hypothesized that earlier components indexing attention allocation, such as the N1, might be also related to predecisional information processing.

In order to study this process, we used the probabilistic inference task (Gigerenzer et al., 1991), modified from its earlier versions (Mata et al., 2007; Wichary et al., 2016) to allow for EEG recording. In terms of behavioral data, we analyzed spontaneous use of decision strategies in this task, as inferred from participants’ actual choices. In the EEG analysis, we looked for differences in the signal amplitude in response to consecutive decision cues that were associated with preference for a particular strategy. We performed this analysis in a relatively novel way, by applying non-parametric cluster based permutation statistics (Maris and Oostenveld, 2007) to single trial analysis with a linear mixed-effects model (Aarts et al., 2014). This approach allowed us to disentangle the contributions of strategy preference and task-related predictors to changes in EEG signal (see Materials and Methods for details) and precisely localize these effects temporally and topographically.

## Materials and Methods

### Participants

Participants were 21 young adult volunteers from Warsaw area. The data of five participants had to be discarded due to artifacts in the EEG signal, therefore the final sample consisted of 16 participants (9 women, age *M* = 23, *SD* = 2.54). Participants provided written informed consent in accordance with the Declaration of Helsinki, under a protocol approved by the Ethics Committee of the Faculty of Psychology, University of Warsaw.

### Apparatus and Materials

#### Decision Task

The computerized probabilistic inference task consisted of making decisions about, which of two diamonds was more expensive. The diamonds were described with the six following cues: size, overall proportions of the diamond, crown proportions, pavilion proportions, size of table and color. The cue values were coded as numbers 0 and 1, with 0 indicating a low value of the cue and 1 indicating a high value. The corresponding cue validities were 0.706, 0.688, 0.667, 0.647, 0.625, 0.6, thus representing a compensatory environment structure (Martignon and Hoffrage, 1999). The cue validities were conditional probabilities of making a correct choice given that the cue discriminated between the alternatives (Rieskamp and Hoffrage, 1999). The cue validities and the cue values for each trial (i.e., the items) were generated by a permutation-based computer simulation using custom made scripts in MATLAB, with the goal to create a stimuli set with a compensatory distribution of cue validities, that maximized the number of discriminative items, that is the items where the choices made WADD and TTB strategies were different. In consequence, this simulation allowed to determine which individual choices were consistent with the WADD rule and the TTB heuristic.

**FIGURE 1:**
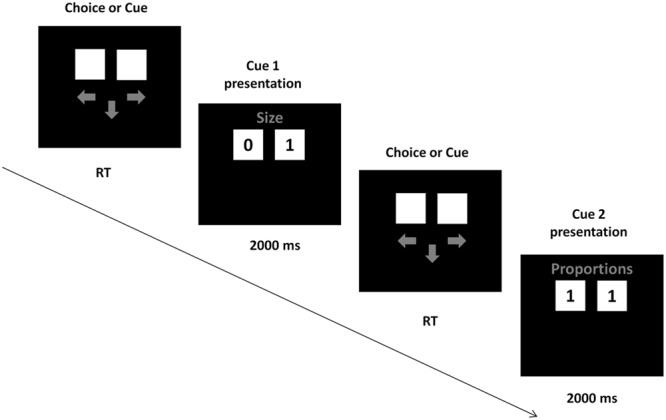
Task presentation. In each trial, participants could either request a cue or make a choice, by pressing the down arrow button (next cue) or left or right arrow buttons (choice). On a given trial, a cue either discriminated between the alternatives (one alternative had a “0” value and the other had a “1” value) or did not discriminate (both alternatives had the same cue value).

The cue validities were presented in the instruction, together with the information that the cues have to be acquired sequentially in the descending order of the validities, from the best cue to the worst. During task performance, participants acquired the cues by pressing the space button on the keyboard with their left hand. SOA between this button press and the cue presentation was 900–1500 ms. After pressing the button, the cue values for both alternatives appeared on the screen for 2s, followed by arrows prompting the participants to either acquire another cue or make the choice. Participants made choices by pressing “arrow left” or “arrow right” buttons on the keyboard with the right hand (**Figure 1**). The task consisted of an instruction phase, a training phase of 3 decisions, and a test phase of 48 decisions. Participants were tested in a separate, dimly lit room. After entering the laboratory, participants were seated ca. 70 cm from the screen, signed an informed consent and provided their demographic data. Afterward, the EEG cap was mounted, participants read the instructions and performed the training and test trials of the choice task.

### EEG Recording and Preprocessing

EEG was recorded using 64-Channel EGI HydroCel^TM^ Geodesic Sensor Net, NetStation software and an EGI Electrical Geodesic EEG System 300 amplifier (Electrical Geodesics, Eugene, OR, United States) with Cz as the reference channel. Input impedance was set below 40 kΩ before the recordings. EEG data were processed using EEGlab (Delorme and Makeig, 2004), Fieldtrip (Oostenveld et al., 2011) and custom-made scripts in MATLAB (Mathworks, Natick, MA, United States). First, EEG signal was high-pass filtered with 1 Hz default EEGlab filter (pop_eegfiltnew function in EEGlab) and cut into consecutive 1-s long windows. Windows further than 1 s away from relevant experimental events (cue presentations) were automatically removed. The remaining windows were visually inspected and those containing strong or non-stereotypic artifacts were removed. Surviving windows were submitted to Independent Component Analysis (ICA). Bad channels (*M* = 1.94; *SD* = 1.68) were not included in the ICA and were later interpolated (see second step below). Independent Components (ICs) representing unambiguous artifacts such as eye movement, eye blinks, heart, or muscle activity were marked for removal.

In the second step the raw unfiltered data were 1 Hz high-pass filtered, the IC weights obtained in the first step were applied to the data and components marked for rejection in the previous step were removed. After clearing the signal from artifacts with ICA, bad channels were interpolated and the signal was 35 Hz low-pass filtered and re-referenced to average. The choice of average reference was motivated by the fact that it reflects well the basic principle that all ERP components are bipolar with both a positive and a negative pole (Dien, 2017). The data were then epoched with respect to cue onsets, yielding epochs starting 250 ms before cue onset and ending 700 ms after cue-onset. Epochs overlapping with data segments that were rejected in the first step of preprocessing were rejected in the second step as well. This procedure – performing ICA on more data than actual epochs was chosen to increase the quality of the decomposition and thus the quality of ICA artifact removal. This two-step approach was implemented in a custom-made, EEGLAB-based toolbox (eegDb toolbox^1^).

### EEG Data Analysis

To compare EEG signal amplitude across all electrodes and in all time samples, we performed a data-driven analysis that does not focus *a priori* on any time-segment or channel group. We used a Monte Carlo based cluster-correction method (Maris and Oostenveld, 2007; Maris, 2012). This approach allows for testing experimental hypotheses at the level of spatio-temporal clusters rather than separate electrodes and provides relevant correction for multiple comparisons. We investigated spatiotemporal patterns of dependency of single-trial EEG signal on the parameters of experimental procedure. To this end, we estimated a linear mixed effects model (LMM) for each time sample and electrode. LMMs take into account the nested structure of the data (like single trials are nested within participants) therefore allowing to include single trials in group analysis. This analysis is less prone to errors of the first and second kind than the traditionally used methods (Aarts et al., 2014). In this model, the following experimental parameters were treated as predictors: (1) cue number (1–6), (2) cue discrimination value (1: cue discriminates vs. 0: cue does not discriminate between the alternatives), (3) relative strategy preference (a continuous variable: the proportion of choices consistent with WADD minus the proportion of choices consistent with TTB). The model also included interaction terms of strategy preference by cue number and strategy preference by cue discrimination value. Participants were entered as random effects, assuming random intercepts, which allowed to account for each participant’s deviation from the average intercept in the model. EEG voltage (standardized within participants’ electrodes) in a time window starting from 200 ms before cue onset and ending at 700 ms after cue onset was treated as the dependent variable. A separate LMM was estimated for each combination of channel and time sample. The model for a specific channel and time sample was captured by the following equation:

$$\begin{array}{c} EEG=\beta_{0}+b_{0i}+\beta_{1}\mathrm{cue}\mathrm{number}+\beta_{2}\mathrm{discriminative}\mathrm{value}+\beta_{3}\mathrm{strategy}\mathrm{preference}+\beta_{4}cue\mathrm{number}*\mathrm{strategy}\mathrm{preference}+\beta_{5}\mathrm{discriminative}\mathrm{value}*\mathrm{strategy}\mathrm{preference}+\varepsilon \end{array}$$

where the EEG signal voltage (at specific channel and time point) is a function of (a) the intercept (β_0_), (b) deviation of given participant from the intercept (*b*_0i_), (c) the cue number (β_1_), (d) the discrimination value of the cue (β_2_), (e) strategy preference (β_3_), (f) interaction of strategy preference and cue number (β_4_), (g) interaction of strategy preference and discrimination value of the cue (β_5_), and (h) the error term 𝜀.

To correct for multiple comparisons, we performed non-parametric cluster-based correction (Maris and Oostenveld, 2007). Cluster correction groups adjacent significant effects (e.g., adjacent time samples on one channel or same time samples on adjacent channels) into continuous clusters. The probability of obtaining stronger effect clusters is evaluated by comparing actual cluster-level statistic (sum of *t*-values within each cluster) to the null distribution of the maximum cluster-level statistic. This null distribution was simulated by permuting the data multiple times and calculating the cluster-level statistics each time. Here, because our model included multiple predictors, each permutation consisted in randomly shuffling predictors between trials within each participant. Subject-level predictor (strategy preference) was shuffled between participants. We performed 1000 permutations where *t*-values for each predictor were clustered separately. Cluster membership threshold was set to >2 *t* and <-2 *t* (negative and positive effects were always clustered separately). Time samples that passed this threshold but did not have a corresponding time sample passing the threshold on any of the neighboring channels were removed from each cluster. This ‘cluster pruning’ was performed to reduce the probability of topographically separate effects being clustered together due to weak single-channel links existing between. Clusters with probability lower than 0.05 (two-tailed) were considered significant. In the second step of the analysis, we focused on P3 and N1 ERP components and showed the differences in these components between participants preferring the complex WADD rule and participants preferring the simple TTB heuristic. EEG data analyses were performed in Julia (Bezanson et al., 2012) with MixedModels.jl package (Bates et al., 2016) and MATLAB (Mathworks, Inc., Natick, MA, United States) with custom-made scripts.

## Results

### Strategy Use and Information Acquisition

**FIGURE 2:**
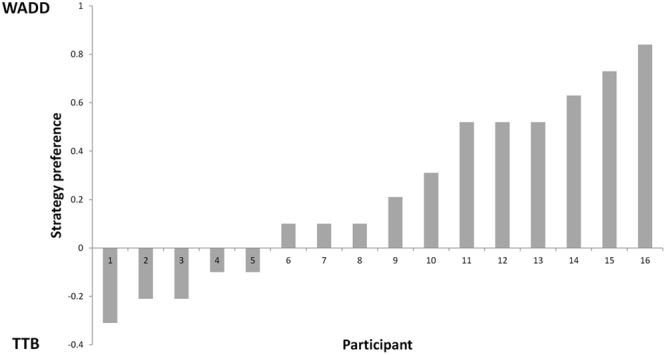
Preference for WADD and TTB strategy across participants.

First, we determined the percentages of choices consistent with each of the two strategies, the Weighted Additive rule (WADD) and the lexicographic heuristic TTB. Across all participants and all trials, WADD predicted 80% of choices and TTB predicted 71.4% choices. Thus, both strategies performed better than chance (50%) in identifying participants’ choices. On discriminating trials (i.e., trials where WADD and TTB made opposite predictions, which constituted 39% of all trials), WADD predicted 60% and TTB predicted 40% of choices, thus WADD was better in explaining participants’ choices. For each participant, we subtracted the proportion of choices consistent with TTB from the proportion of choices consistent with WADD (**Figure 2**). We used this single continuous variable (the relative preference of WADD over TTB) in the subsequent analysis. To characterize participants’ pre-choice behavior, we quantified their information search by counting how many cues a participant requested before each choice. On average participants acquired 4.7 cues (out of 6), and there was no correlation between individual preference for strategy and the number of acquisitions (*r* = 0.28, *p* = 0.34). This is in contrast to some behavioral studies on decision strategy use (e.g., Rieskamp and Hoffrage, 2008; Wichary et al., 2016), however, it can be explained by the fact that cue acquisition in our task was costless and easy, and the compensatory structure of the task environment (the cues had similar validities) favored the acquisition of all the available cues (see Newell and Shanks, 2003 for similar results). Participants’ total decision time was on average 4540 ms (*SD* = 2023 ms), and it did not significantly correlate with individual strategy preference (*r* = -0.44, *p* = 0.09; see Supplementary Material for the performance of individual participants).

### EEG Results

We analyzed EEG signal amplitude in response to consecutive decision cues, to determine whether strategy preference was associated with EEG signal amplitude. As mentioned earlier, we estimated a LMM for each time sample and electrode with the following predictors: (1) cue number (1–6), (2) cue discrimination value (1: cue discriminates vs. 0: cue does not discriminate between the alternatives), (3) relative strategy preference (a continuous variable: the proportion of choices consistent with WADD minus the proportion of choices consistent with TTB), and EEG voltage in a time window starting from 200 ms before cue onset and ending at 700 ms after cue onset was treated as the dependent variable.

**FIGURE 3:**
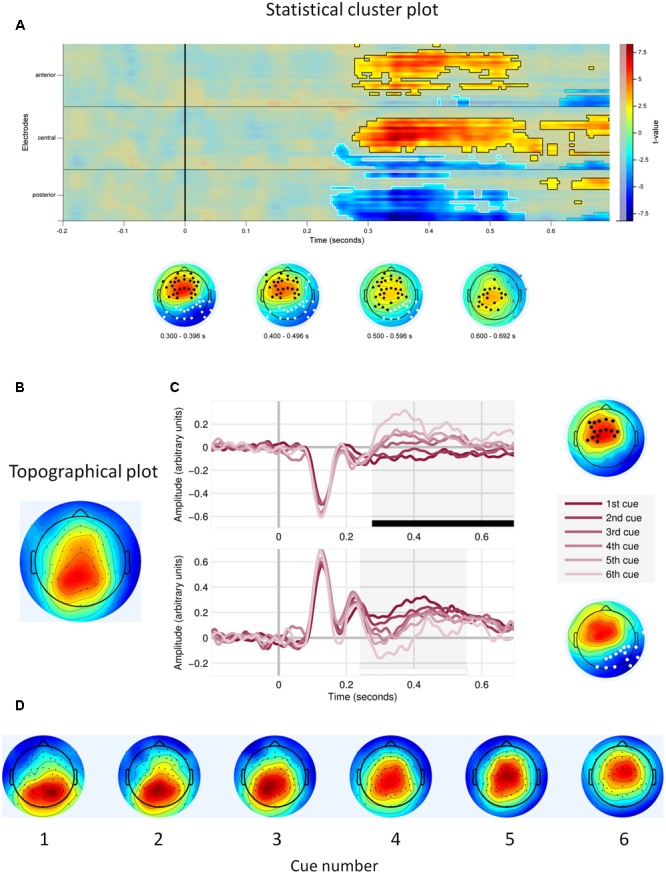
Main effects of cue number. **(A)** Statistical cluster plot for the cue number effect with significant electrodes, scalp and time location. **(B)** Topographical plot of the P3 component (time window 300–400 ms), averaged across participants, trials and cues. **(C)** Event related potentials in response to each decision cue, at the frontal cluster (Upper) and at the parietal cluster (Lower). **(D)** Topographical plots of P3 responses (time window 300–400 ms) to consecutive decision cues, averaged across participants and trials.

First, we observed main effects of cue number on the EEG signal amplitude. **Figure 3A** shows that the effects were located in two electrode clusters – a midline fronto-central cluster and a posterior, parieto-occipital cluster, within the time window 250–700 ms. EEG signal amplitude on frontal and central electrodes within that window increased with cue number (*p* < 0.001, cluster-corrected). Given our interest in P3, we focus here on the posterior cluster, where this component is most often located and where we also observed it in the current study (**Figure 3B**). At this cluster, the signal amplitude was negatively related to the cue number – it was highest to the first (most important) cue and decreased with each consecutive cue (*P* < 0.0001; **Table 1** and **Figure 3C**). In addition, topographical plots of the EEG voltage within the P3 time window showed a changing scalp topography of this component (**Figure 3D**).

**Table 1:** Model estimates for the effects of task characteristics and individual strategy preference on EEG amplitude.

Effect	Electrode cluster	Time range (post-stimulus)	Estimate (cluster level sum of *t*-values)	*P* (permutation-based, cluster-corrected)
Cue number (1–6)	Positive	276–696 ms	6171.99	<0.001
	Negative 1	240–556 ms	–4585.34	<0.001
	Negative 2	616–696 ms	–621.02	0.034
Cue discriminates (ref: 0)	Positive	316–600 ms	2878.66	<0.001
	Negative 1	332–476 ms	–1320.00	0.004
	Negative 2	548–680 ms	–780.01	0.018
Strategy preference (WADD-TTB)	Positive	304–472 ms	1131.88	0.008
Strategy preference:cue number	Positive	336–480 ms	1158.71	0.010
	Negative	336–528 ms	–1762.52	<0.001
Strategy preference:cue discriminates	Positive 1	404–580 ms	1253.00	0.002
	Negative 1	408–500 ms	–836.68	0.012
	Positive 2	124–224 ms	589.99	0.04
	Negative 2	120–192 ms	–523.80	0.038

*WADD (TTB) – proportion of choices consistent with Weighted Additive strategy. (Take The Best heuristic). The estimate we provide (cluster-level sum of *t*-values) depends both on cluster size and cluster strength.*

**FIGURE 4:**
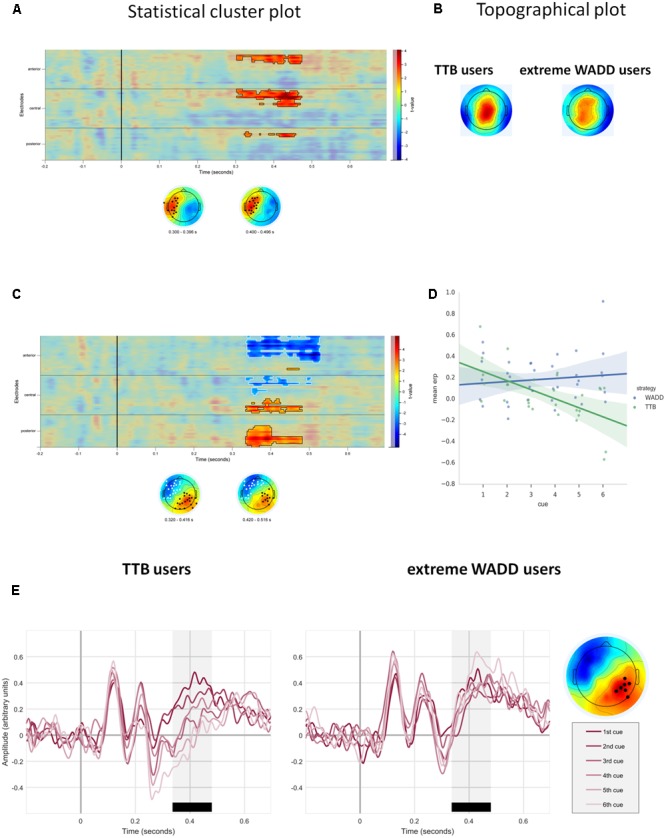
**(A)** Temporal and scalp distribution of the main effect of strategy preference on EEG signal amplitude. **(B)** Topographical plots of the standardized voltage of the P3 component, averaged across trials and cues, for the groups of TTB users and extreme WADD users. **(C)** Temporal and scalp distribution of the cue number and strategy preference interaction effect. **(D)** Distribution of averaged EEG amplitudes to decision cues for TTB users and extreme WADD users, at the parietal electrode cluster within the time window 350–450 ms. **(E)** ERPs to decision cues plotted for the TTB users and extreme WADD users at the parietal cluster.

Importantly, we observed a main effect of strategy preference. This positive effect was located on the frontal, central and some parietal electrodes on the left side of the scalp. Higher preference for the WADD strategy was associated with higher signal amplitude to decision cues in this cluster, within the time window from 300 to 470 ms (*P* = 0.008; **Table 1** and **Figures 4A,B**). We also observed an interaction effect of strategy preference and cue number, indicating that individuals preferring WADD and TTB strategies processed the consecutive cues differently. **Figure 4C** shows that this effect was located at two electrode clusters – a negative effect on left frontal electrodes (*P* < 0.001) and a positive effect on right parietal electrodes (*P* = 0.01). Given our interest in the P3 component, we focus here on the parietal cluster, where this component is usually observed and where we also observed it in our study (**Figure 3B**). Although the analysis was performed with strategy preference as a continuous predictor, for illustration purposes, we divided the participants into tertile groups and plotted the ERPs and their averages separately for two extreme groups: the “TTB users” and the “extreme WADD users.” For the TTB users, the majority of their choices were made with the TTB heuristic, whereas for WADD users, the majority of their choices was made with the WADD rule. For TTB users, the signal amplitudes to the consecutive cues within the P3 time window decreased with each consecutive cue, while for the extreme WADD users, the amplitudes were similar (**Figures 4D,E**).

**FIGURE 5:**
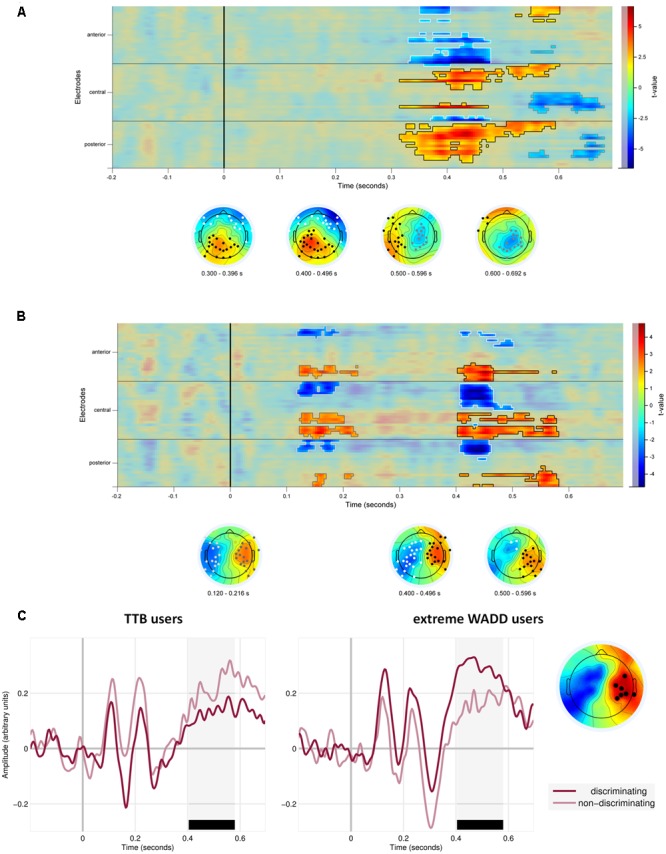
**(A)** Temporal and scalp distribution of the main effect of cue discrimination. Marked in black, white and gray are the spatiotemporal clusters where the EEG amplitude differed for the discriminating and non-discriminating cue. **(B)** Temporal and scalp distribution of the interaction effect of discriminative value and strategy preference. **(C)** ERPs to the discriminating and non-discriminating cues plotted for TTB users, and extreme WADD users. The time window where the effects in the P3 range were observed is marked in black.

We also observed effects involving the discrimination value of the cue. First, on average, EEG signal amplitude differed in response to discriminating cues than in response to the non-discriminating cues (the main effect of the discriminative value of the cue; **Figure 5** and **Table 1**). **Figure 5A** shows the statistical cluster plot of these effects, a positive effect (*P* < 0.0001) at a parietal cluster, two negative effects (*P* = 0.004, *P =* 0.018), at a frontal and central cluster in the time window between 300 and 650 ms. On the parietal cluster within the P3 window, which is our main focus, signal amplitude was higher in response to discriminating cues than in response to the non-discriminating cues.

We also observed interaction effects of the discriminative value of the cues and strategy preference, indicating that strategy preference modulated the processing of discriminating and non-discriminating cues. These effects were present at four electrode clusters, two within an early time window of 120–220 ms and two within a later window of 400–600 ms (**Figure 5B**).

In the 400–600 ms time window corresponding to P3, a negative effect (*P* = 0.012) was present at central and parietal electrodes on the left side and a positive effect (*P* = 0.002) was present at central and parietal electrodes on the right side. Focusing on the right central and parietal electrode cluster, we found that high preference for WADD was associated with higher EEG amplitudes to discriminating cues than to non-discriminating cues, and high preference for TTB was associated with lower amplitudes to discriminating cues than to non-discriminating cues (**Figure 5C**).

Interestingly, in the 120–220 ms range, corresponding to the N1 component, a negative effect (*P* = 0.038) was present at frontal, central and parietal electrodes on the left side and a positive effect (*P* = 0.04) at the analogous electrodes on the right side (**Figure 5B**). High preference for TTB was associated with lower EEG amplitudes (i.e., with more pronounced N1) to discriminating cues than to the non-discriminating cues (**Figure 5C**). This was evident at the right central electrode cluster.

In sum, our analysis provided evidence that the individual variability in the use of WADD and TTB strategies is associated with differences in EEG signal amplitudes to decision cues, most prominently within the time range of the P3 component, with additional effects on the N1 component.

## Discussion

What are the neural correlates of normative and heuristic decision strategies? To answer this question, we conducted an EEG study where participants performed multi-attribute probabilistic inference task and could use different decision strategies to utilize the available information. Most participants integrated the acquired the pieces of information into the decision process by using the normative Weighted Additive rule on the majority of the trials, as indicated by their choice outcomes. Some participants, however, did not integrate the acquired cues into the final choice, relying instead on a simple heuristic TTB on most of the trials.

We found that, on average, the relative preference for the WADD strategy was associated with higher P3 amplitudes to decision cues on the left frontal, central and parietal electrodes. From the perspective of P3 as an index of endogenous attention, this result suggests that participants preferring the complex WADD strategy could better mobilize attentional resources in response to the decision cues than the participants preferring the simple heuristic TTB. Another possible, and not mutually exclusive interpretation of this result is that participants preferring WADD had higher processing capacity, manifested in higher P3 amplitudes. This is consistent with findings by Nittono et al. (1999) and Gevins and Smith (2000) who showed that individuals with high working memory span exhibit larger P3 responses to stimuli in working memory tasks. Also, the scalp location of this effect suggests that the observed differences might originate from differences in working memory capacity. For example, Öztekin et al. (2009) and Polanía et al. (2012a) showed the involvement of left frontal and parietal regions in working memory, and Polanía et al. (2012b) showed that transcranial alternating current brain stimulation at left frontal and parietal sites improves working memory functioning. In this perspective, our findings suggest that the preference for the complex WADD strategy might originate from the high ability to hold the incoming decision cues in working memory and combine them into the final choice.

We also found that the signal amplitude in the P3 window differed for each consecutive cue. At midline frontal cluster, the signal amplitude increased with each consecutive cue. This might reflect the effects of increasing cognitive load, similar to those reported in other EEG studies, where ERP and time-frequency analyses showed the effects of load (Onton et al., 2005; Gomarus et al., 2006). At the posterior cluster, overlapping the parietal electrodes where we located the P3 component and where it is analyzed in many other studies, the signal amplitude to consecutive cues decreased, possibly indexing decreasing orienting response toward the incoming cues. Also, topographical plots of the voltage in the P3 time window indicated that with each consecutive cue, the maximum voltage of this component was located more frontally. This changing topography of the ERP could at least partially explain both its decreases at the parietal electrodes and the increases at the frontal electrodes. These results together might reflect the interplay between the executive network vs. salience network changing with increasing working memory load (e.g., Seeley et al., 2007; Menon and Uddin, 2010), however, given the lack of source analyses in our current results, we cannot provide definitive answers here.

Importantly, strategy preference moderated the effect of cue number, so that the high preference for WADD was associated with similar P3 amplitudes to consecutive cues, whereas preference for TTB was associated with largely different P3 amplitudes to the consecutive cues, with substantial decreases from the best cue to the worst. This result suggests that individuals preferring WADD and TTB strategies differ in the endogenous attentional weighting of the consecutive decision cues, with TTB users highly weighting the first cue and weighting the following cues substantially less, and WADD users weighting all cues similarly. In this perspective, the P3 component at the parietal electrodes can be interpreted as indexing endogenous attentional weighting of decision cues that shapes predecisional information processing and strategy use in multi-attribute choice.

We also found that strategy preference moderated the effect of the discriminative value of the cues on the ERPs to the cues. High preference for WADD was associated with higher P3 to discriminating than to non-discriminating cues. In contrast, high TTB preference was associated with lower P3 amplitudes to the discriminating cues than to the non-discriminating cues. However, we also observed the association between strategy preference and EEG responses to discriminating vs. non-discriminating decision cues in the latency range of the N1 component. Particularly, TTB users showed a large N1 to the discriminating cues while showing a small P3 to these cues. The N1 is interpreted as an early attentional component, indexing automatic allocation of sensory attention (Näätänen and Picton, 1987; Vogel and Luck, 2000). Together, these findings might constitute evidence for the association of the preference for TTB with an early automatic attention allocation to discriminating cues, but also relatively low engagement of endogenous attention processes to these cues.

To our knowledge, this is the first study that shows the relationship of decision strategy preference in inference-based multi-attribute choice with characteristics of the EEG signal. Earlier studies of strategy use in this paradigm (Venkatraman et al., 2009a,b; Khader et al., 2011; Gluth et al., 2013) employed fMRI and thus could not track the fine grained temporal dynamics of brain activity underlying strategy use. Here, we show that the individual variability in strategy use is associated with differences in early processing of the decision cues prior to making choices, which is indexed by the amplitudes of the P3 and N1 ERP components. In one study on strategy use in probabilistic inference that relied on ERP analysis, Rosburg et al. (2011) showed that P3 amplitude predicted the use of the simple recognition heuristic. However, in that study no alternative strategies were analyzed. We go beyond Rosburg’s et al. (2011) focus on the recognition heuristic by looking at alternative strategies that represent two extremes, the normative Weighted Additive rule and the simple heuristic TTB, and we show that the P3 component can be associated with strategy use even beyond the recognition heuristic.

The P3 component reflects phasic changes in arousal in response to changes in the environment (Polich, 2014). It indexes the allocation of attentional resources to relevant stimuli, with more motivationally significant stimuli eliciting larger P3 amplitudes. Our results are in line with this interpretation. P3 recorded at parietal electrodes, where it is usually detected, was highest to the most important cues and decreased for each consecutive cue. Moreover, for participants preferring the TTB heuristic this decrease was relatively large, whereas for WADD users the P3 amplitudes to the consecutive cues were similar. This suggests that the preference for the WADD vs. TTB strategy can be explained, at least partially, by the differences in the allocation of attention to decision cues. Moreover, the preference for WADD strategy was associated, on average, with higher P3 amplitudes to decision cues. In line with the attention allocation argument, it suggests that the preference for WADD is associated with a greater ability to mobilize attentional resources in response to any cue. Enhanced attention to stimuli and the associated high P3 amplitudes predict stimuli encoding (Polich, 2014). Together, these findings suggest that the preference for WADD was caused by greater attention to all cues and their better representation in memory.

It has been proposed that P3 is modulated by the activity of the LC-NE (Nieuwenhuis et al., 2005). Differences in P3 amplitude are thought to be the effect of the modulation of information processing by LC-NE controlling the gain of activation in the cortex (e.g., Eldar et al., 2013), which is supported by the evidence in Murphy et al. (2011, 2014). Adopting this perspective, we suggest that the preference for a particular choice strategy is at least partly determined by the LC-NE gain control mechanism. Our theoretical model of decision strategy selection (Wichary and Smolen, 2016) assumes that phasic gain changes in response to decision cues constitute the mechanism of differential attentional weighting of decision cues during option evaluation, and thus shape the strategy for pre-decisional information processing. Norepinephrine might be one neuromodulator responsible for the observed effects, however, P3 amplitude was also linked with activity in ventral striatum (Pogarell et al., 2011; Pfabigan et al., 2014), suggesting that also dopamine is involved in the modulation of this component. This is consistent with early views by Servan-Schreiber et al. (1990) and Arnsten (1998), suggesting similar effects of dopamine and norepinephrine on attention and working memory (see also Robbins and Arnsten, 2009). Thus, a broader interpretation of our results is that the activity of both catecholamine systems modulates information processing in the cortex so as to prioritize content in working memory. In the context of multi-attribute decision making, variation in this activity leads to choices consistent with different decision strategies.

Regarding the N1, we showed the association between strategy preference and EEG responses to discriminating vs. non-discriminating decision cues. A mechanistic explanation of the observed effects could take into account that the N1 component has been linked to mental effort invested in stimulus processing, indexed by the activation of ACC. Mulert et al. (2008), in an fMRI-EEG study, showed that N1 amplitude to relevant stimuli was highly correlated with ACC activation during effortful, perceptual decision making. In this perspective, the association of high N1 amplitude to discriminating cues with TTB preference suggests that individuals preferring this choice strategy invest cognitive effort into processing such cues very early during the decision process.

We obtained our results using a more advanced method than the traditional ERP analysis, which allowed us to precisely localize the effects in time and on the scalp. It is worth noting that nearly all effects we detected follow a dipolar pattern suggesting that the pairs of negative and positive effects were generated by spatially distinct neural sources. However, since we did not perform source analysis on the current data, we cannot provide answers about the actual neural sources of the observed effects. Nevertheless, given the topographic distribution of the ERPs, we could focus our analysis on the electrode clusters that encompass the electrodes usually analyzed in the context of P3 component, and we could show the differences in the amplitude of this component in response to decision cues that were related to decision strategy preference, as identified on the basis of choices.

It is important to note that the strategy preference was not related to the number of acquired decision cues in our study. While it may seem surprising at first, and several studies show an association of strategy preference with information acquisition (e.g., Rieskamp and Hoffrage, 2008; Wichary et al., 2016), others (e.g., Newell and Shanks, 2003, see Bröder and Newell, 2008 for discussion) have shown that participants using simple heuristic often acquire more information that is required by the heuristic, even if the information acquisition is costly (e.g., there is monetary cost of acquiring cues). In our study, strategy preference was not associated with the number of acquired cues, therefore the analysis of information acquisition alone would not allow us to distinguish between participants preferring WADD or TTB. In contrast, the analysis focused on choice outcomes coupled with EEG recording, allowed us to elucidate the fate of the acquired information that is if it was integrated into the decision process and how it was processed.

Although we analyzed the effects of behavioral strategy preference as a continuous predictor, we observed its effects on a relatively small sample and in a particular (compensatory) task environment. Therefore, a question remains open whether such associations will generalize to larger samples with higher variability in strategy preference (i.e., with more choices consistent with TTB heuristic) and in tasks with a non-compensatory information structure. Since the current study was aimed at finding correlates of individual differences in spontaneous strategy use in a complex decision making task, we focused on the compensatory task environment. However, task information structure is an important source of variability in strategy use and its impact on EEG correlates should be addressed in future studies. Also, a question remains whether these effects generalize to studies with situational factors known to impact strategy preference, such as time pressure (Rieskamp and Hoffrage, 2008) or emotional arousal (Wichary et al., 2016).

## Conclusion

Humans use heuristics to overcome the bounds of rationality (Gigerenzer et al., 1999). Individual variability in decision strategy use is a central research problems in this area (Ford et al., 1989; Pachur and Bröder, 2013) and our study helps to understand the sources of this variability by employing EEG as a fine grained method to analyze the dynamics of pre-decisional information processing and choice. It provides initial evidence that the P3 and N1 ERP components indexing attention allocation are associated with the use of rational and heuristic choice strategies and can be further studied as early correlates of strategy preference.

## Author Contributions

SW conceived the research. SW, TO, and AB planned the study. TO conducted the study under the supervision of SW and AB. SW, MM, and TO processed and analyzed the data. SW and MM wrote the manuscript and TO and AB revised the manuscript.

## Conflict of Interest Statement

The authors declare that the research was conducted in the absence of any commercial or financial relationships that could be construed as a potential conflict of interest.

## Footnotes

1 https://github.com/mmagnuski/eegDb
