# Supplementary material for: Neural Signatures of Rational and Heuristic Choice Strategies: A Single Trial ERP Analysis
Source: Front Hum Neurosci. 2017 Aug 18;11:401. doi: 10.3389/fnhum.2017.00401 (PMC5563328; doi:10.3389/fnhum.2017.00401)
Supplement: Supplementary file 1 [file Data_Sheet_1.docx]

**Neural signatures of rational and heuristic choice strategies:**

**A single trial ERP analysis**

Szymon Wichary^1^, Mikołaj Magnuski^2^, Tomasz Oleksy^3^, Aneta Brzezicka^2,4^

^1^Wrocław Faculty of Psychology, University of Social Sciences and Humanities, Wrocław

^2^Faculty of Psychology, University of Social Sciences and Humanities, Warsaw

^3^Faculty of Psychology, University of Warsaw

^4^Department of Neurosurgery, Cedars-Sinai Medical Center, Los Angeles, USA

Corresponding author:

Szymon Wichary, PhD

Center for Research in Economic Behavior

Wrocław Faculty of Psychology

SWPS University of Social Sciences and Humanities

Ul. Ostrowskiego 30b, 53-238 Wrocław, Poland

Email: swichary@swps.edu.pl

**Supplementary Information**

Method

*EEG data analysis*

Here, we provide the specifications of the cluster-based analysis.
*Definition of neighbours.* Neighbours were defined based on the channel position. The procedure was performed with Fieldtrip’s ft_prepare_neighbours and the obtained mesh was later hand-corrected. ft_prepare_neighbours first projects channels to 2d space and then uses Delaunay triangulation to construct a mesh connecting the channels. This mesh is then hand corrected to exclude unnecessary connections or add missing ones. We add a few snapshots of our adjacency mesh below (Fig. SI1).


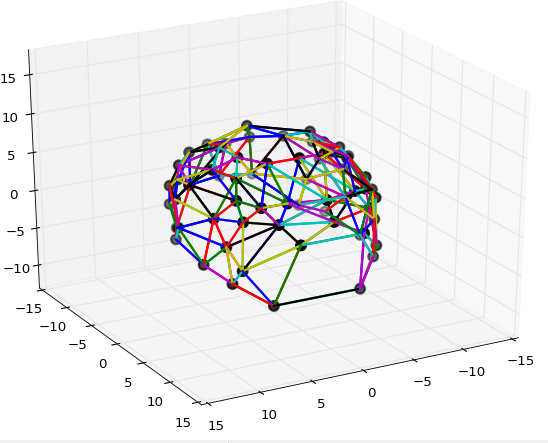

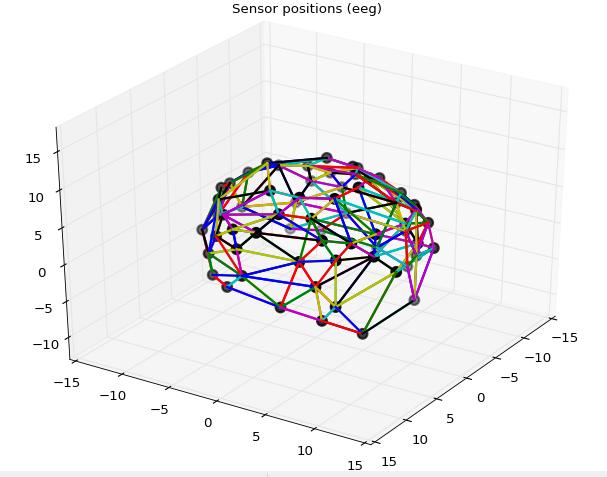


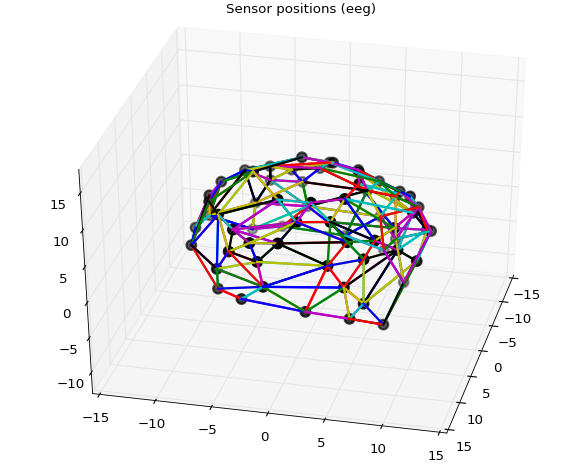

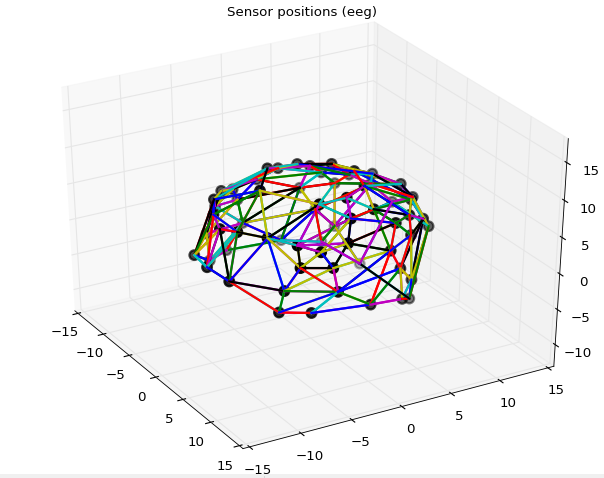


Figure S1. Snapshots of the adjacency mesh used in the data analysis.

*Number of clusters and cluster definition.* There was no predefined number of clusters, the clusters were created from spatio-temporally adjacent significant effects and then nonparametrically corrected for multiple comparisons at the cluster-level. Cluster definition was standard - samples that are adjacent and significant are grouped into clusters and their t-values are summed.

*Min/max number of adjacent electrodes for a sample to enter a cluster.* This parameter works like a low-pass filter on the clusters and thus prevents single-channel bridges between separate clusters. Such bridges would cause the clustering algorithm to consider these clusters as one cluster. The value of 2 (cfg.minnbchan = 2) for this parameter is standard - which is reflected be the fact that it is used in fieldtrip’s official clustering tutorial (<http://www.fieldtriptoolbox.org/tutorial/cluster_permutation_timelock>).

*Electrode regions.* We used all channels, except for cheek electrodes that manifest many artifacts and the Cz channel that was used as the reference channel and was removed during re-referencing to average.

Results

Table S1

*Participants’ behavioral performance.*

| *Participant* | *Strategy preference* | *Strategy preference tertile* | *Classification* | *Mean number of acquired cues* | *Mean decision time (ms)* |
| --- | --- | --- | --- | --- | --- |
| 1 | -0.31579 | 1 | TTB user | 4.72 | 3657 |
| 2 | -0.21053 | 1 | TTB user | 5.54 | 8795 |
| 3 | -0.21053 | 1 | TTB user | 4.75 | 7940 |
| 4 | -0.10526 | 1 | TTB user | 4.77 | 6692 |
| 5 | -0.10526 | 1 | TTB user | 4.2 | 4200 |
| 6 | 0.105263 | 2 | moderate WADD user | 5.5 | 2794 |
| 7 | 0.105263 | 2 | moderate WADD user | 5.1 | 5122 |
| 8 | 0.105263 | 2 | moderate WADD user | 4.25 | 3202 |
| 9 | 0.210526 | 2 | moderate WADD user | 4.68 | 4140 |
| 10 | 0.315789 | 2 | moderate WADD user | 3.42 | 2902 |
| 11 | 0.526316 | 3 | extreme WADD user | 4.75 | 4686 |
| 12 | 0.526316 | 3 | extreme WADD user | 5.22 | 2244 |
| 13 | 0.526316 | 3 | extreme WADD user | 3.7 | 4075 |
| 14 | 0.631579 | 3 | extreme WADD user | 4.77 | 2836 |
| 15 | 0.736842 | 3 | extreme WADD user | 5.35 | 6885 |
| 16 | 0.842105 | 3 | extreme WADD user | 4.54 | 2477 |
